# Supplementary material for: Targeting transcription in heart failure via CDK7/12/13 inhibition
Source: Nat Commun. 2022 Jul 27;13:4345. doi: 10.1038/s41467-022-31541-8 (PMC9329381; doi:10.1038/s41467-022-31541-8)

A

Cancer Cell Lines	THZ1 GI50 (nM)
Diffuse intrinsic pontine glioma cells (Nagaraja et al., 2017)	28 to 533
Oesophageal squamous cell carcinoma cell lines (Jiang et al., 2017)	21 to 192
Mouse small cell lung cancer cell lines (Christensen et al., 2014)	75 to 100
MYCN-amplified nuroblastoma cell lines (Chipumuro et al., 2014)	< 10
Peripheral T-cell lymphomas cell lines (Cayrol et al., 2017)	250 to 500
Ovarian Cancer cell lines (Francavilla, C., 2017)	5 nM to 300
Patient-derived primary high-grade glioma cell lines (Greenall et al., 2017)	< 100
Breast cancer cell lines (Harrod et al., 2017)	160 to 190
Triple-negative breast cancer cell lines (Li et al., 2017)	270 to 440
Nasopharyngeal carcinoma cell lines (Yuan et al., 2017)	100 to 1000
Osteosarcoma cell lines (Chen et al., 2018)	< 75
Cutaneous melanoma cell lines (Eliades et al., 2018)	18 to 45
Ewing sarcoma cell lines (Iniguez et al., 2018)	24 to 47

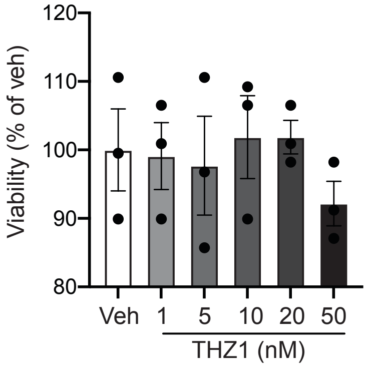

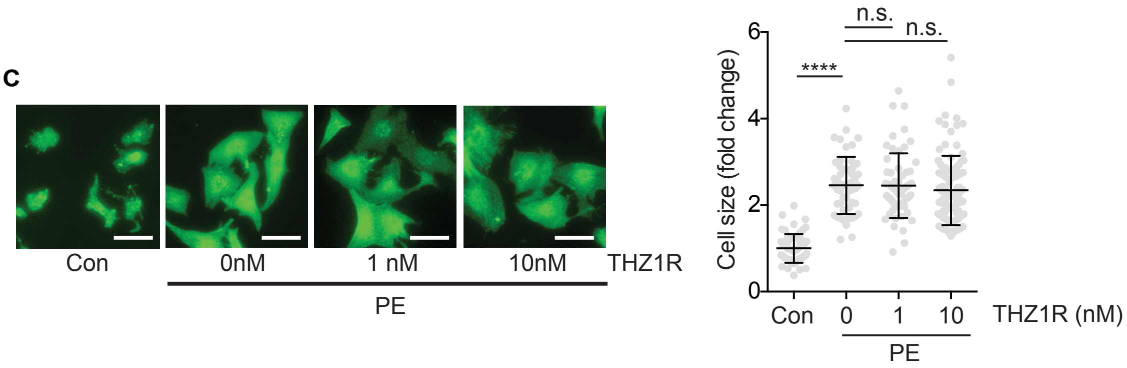

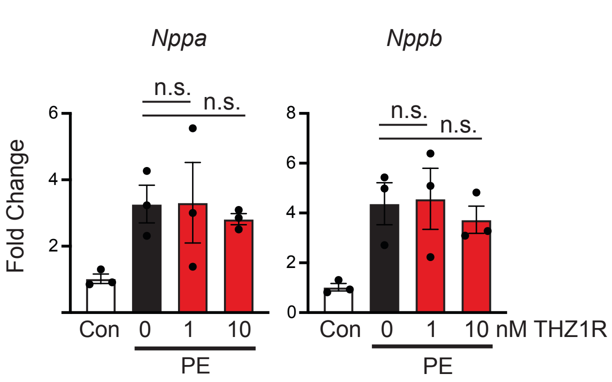

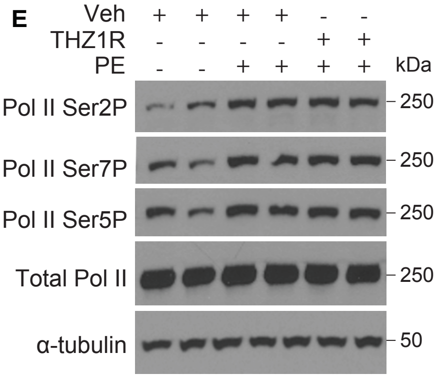

Supplementary Figure 1. Effects of THZ1 and THZ1-R. (A) Summary of THZ1 concentrations causing 50% cell growth inhibition (GI_{50}) for indicated cancer cell types. These data have been curated from published literature and summarized here. **(B)** Trypan blue staining for indicated doses of THZ1 (n=3). **(C)** Representative NRVM image (α-actinin in green) and quantification of cell size with indicated treatments (n=59 cells for control, n=51 cells for PE, n=58 cells for THZ1R-1nM + PE, n=100 cells for THZ1R-10nM + PE). Scale bar: 30 μm. Bars denote mean ± SD. **(D)** Quantification of expression levels of representative genes (*Nppa* and *Nppb*) with indicated treatments (n=3). **(E)** Representative Western blots of NRVM with indicated treatments for specific targets. Pol II phosphoforms and total Pol II were each probed from a separately run gel and membrane, with each lane loaded with an equivalent amount of total protein and lysate volume, derived from a common master stock of protein lysate for each designated condition. The loading control (α-tubulin) was probed on the same blot with Pol II Ser7P. For these Western blots, NRVM were harvested 30 min after exposure to PE. Experiment was repeated three independent times with similar results.****$p < 0.0001$, not significant (n.s., $p > 0.05$) for indicated comparisons. Data are shown as means ± SEM unless otherwise noted. One-way ANOVA with Tukey's multiple comparisons test was used for all statistical analyses. Exact p values are noted when possible. Source data are provided as a Source Data file.

Supplementary Figure 2

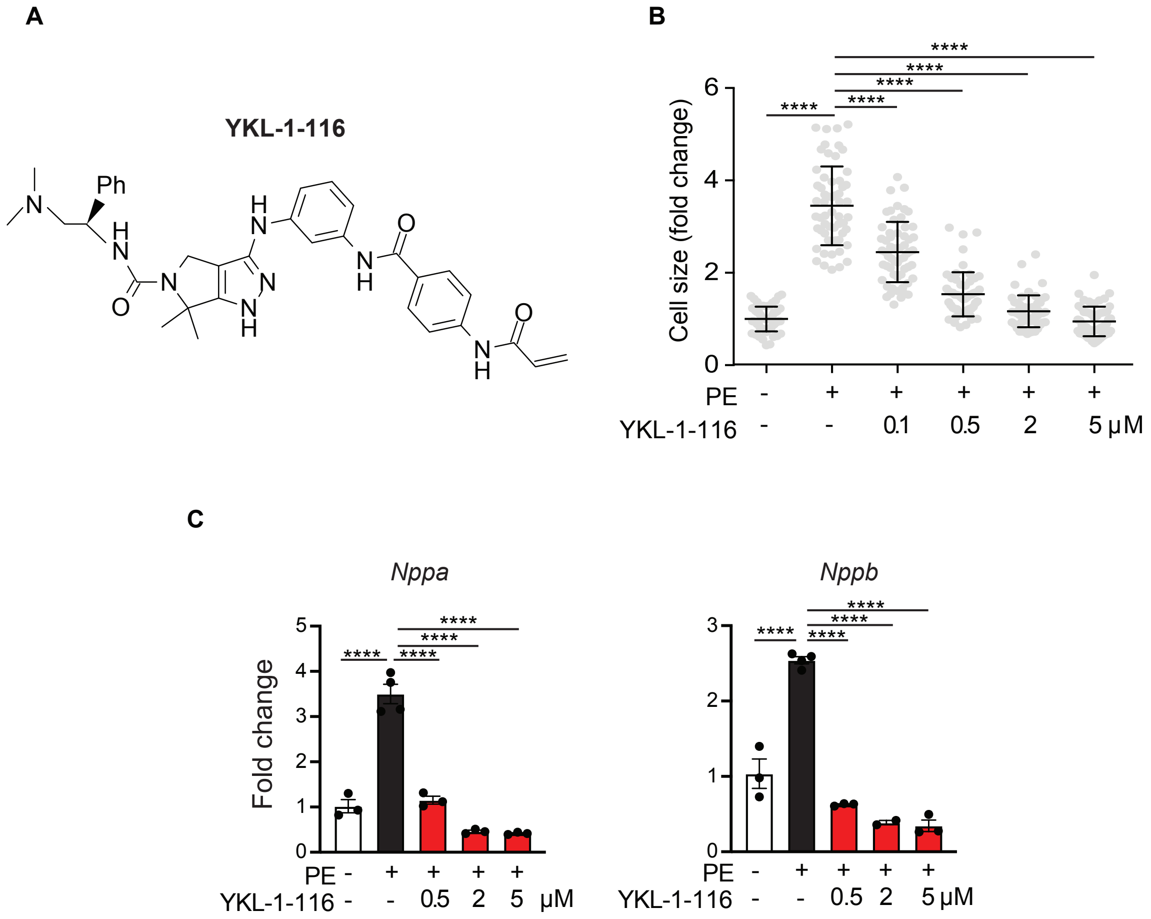

Supplementary Figure 2. YKL-1-116 inhibits agonist-induced hypertrophy in NRVM. (A) Chemical structure of YKL-1-116. **(B)** Quantification of cell area with indicated treatments (n=55-58 randomly selected cells per condition). Bars denote mean ± SD. **(C)** Gene expression levels of representative genes (*Nppa* and *Nppb*) in NRVM with indicated treatments (n=3-4; for *Nppb*, the YKL 2μM group is n=2). Bars denote mean ± SEM. ****$p < 0.0001$ for indicated comparisons. One-way ANOVA with Tukey's multiple comparisons test was used for all statistical analyses. Exact p values are noted when possible. Source data are provided as a Source Data file.

Supplementary Figure 3

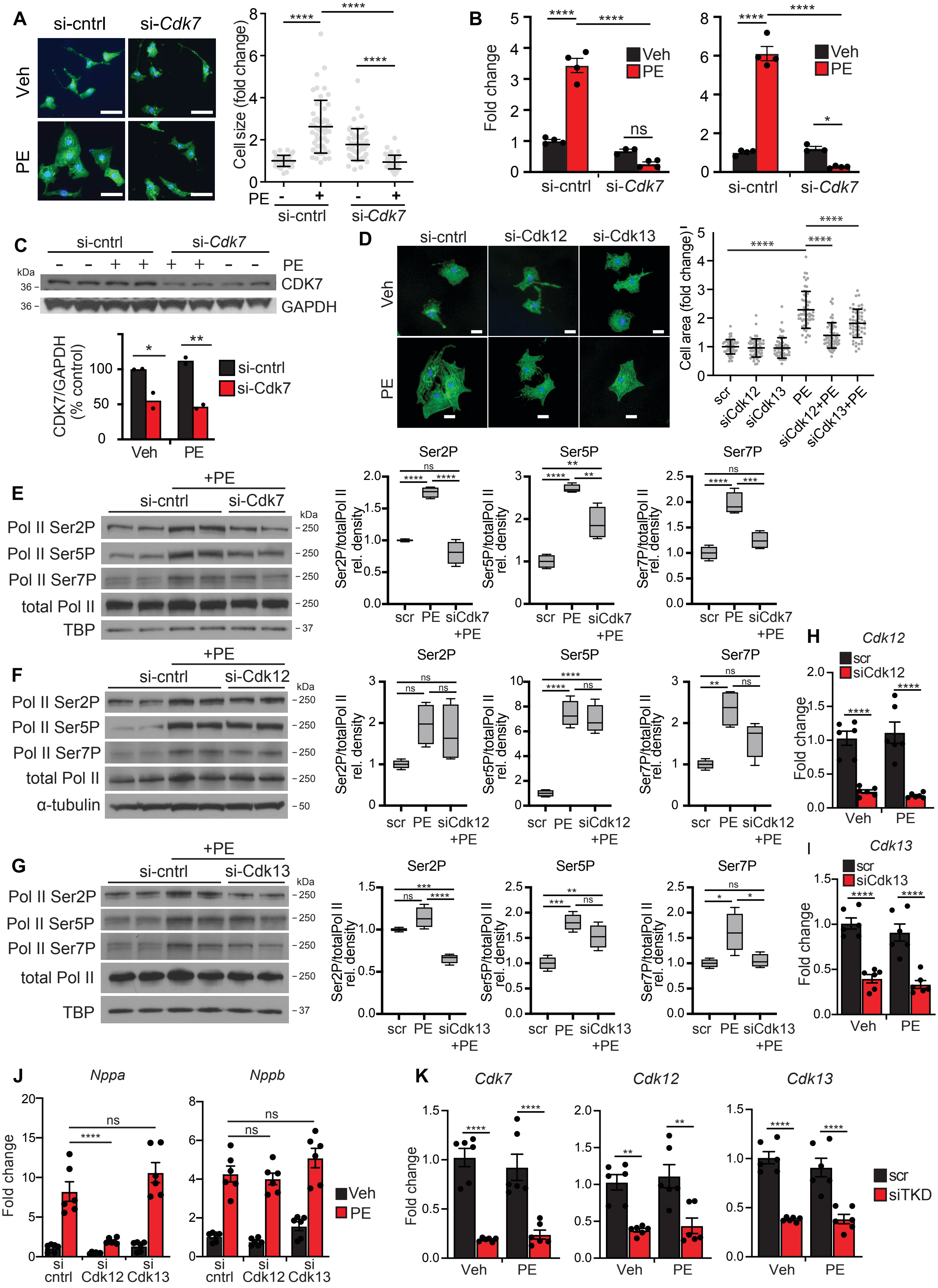

Supplementary Figure 3. Effects of si-*Cdk7*, si-*Cdk12,* si-*Cdk13,* and si-TKD (triple knock down) (A) Representative images of NRVM treated ± si-control (si-cntrl) or si-*Cdk7* and PE (100 μM) for 48h with cell area quantification (n=40 cells for si-cntrl, n=51 cells for si-cntrl+PE, n=57 cells for si-Cdk7, n=53 cells for si-Cdk7+PE). Scale bar: 30 μm. Bars denote mean ± SD. **(B)** Quantification of expression levels of indicated genes from real-time PCR (n=3-4). Scale bar: 30 μm. **(C)** Representative western blot of NRVM with indicated treatments for the specific targets and quantification of CDK7/GAPDH ratio (n=2). The loading control (GAPDH) was probed on a separate blot from CDK7. Experiment was repeated three independent times with similar results. **(D)** Representative images of NRVM treated ± si-control (si-cntrl), si-*Cdk12*, or si-*Cdk13* and ±PE (100 μM) for 48h with cell area quantification (n=55 randomly selected cells per condition). Scale bar: 30 μm. Bars denote mean ± SD. The experiment for single knockdown of *Cdk12*±PE and *Cdk13*±PE shown here was performed as part of the same experiment for triple knockdown (TKD) of *Cdk7/12/13*±PE that is shown in main Figure 1E. Therefore, these experiments share the exact same control groups for si-cntrl±PE. The representative image and area quantification for the si-cntrl±PE conditions are duplicated here from Figure 1E, as they appropriately serve as the same control groups for these two figures. The panels for triple knockdown were separated and placed into the main Figure 1E to provide clarity during the description of the Results section. **(E-G)** Representative Western blots of NRVM with indicated treatment for specific targets. Quantification is shown on the right (N=4) with data shown as box plots with error bars representing SD. Pol II phosphoforms and total Pol II were each probed from a separately run gel and membrane, with each lane loaded with an equivalent amount of total protein and lysate volume, derived from a common master stock of protein lysate for each designated condition. The loading control (TBP or α-tubulin) was probed on the same blot with Pol II Ser7P. For these Western blots, NRVM were harvested 30 min after exposure to PE. Box plots show center line as median, whiskers show maxima and minima, and box limits show upper and lower quartiles. **p = 0.0017 for siCdk7 (Ser5P): Scr vs. siCdk7 + PE, **p = 0.0033 for siCdk7 (Ser5P): PE vs siCdk7 + PE, ***p = 0.0008 for siCdk7 (Ser7P): PE vs siCdk7 + PE. **p = 0.0016 for siCdk12 (Ser7P): scr vs PE, ***p = 0.0005 for siCdk13 (Ser2P): scr vs. PE, ***p = 0.0004 for siCdk13 (Ser5P): scr vs PE, **p = 0.0067 for siCdk13 (Ser5P): scr vs siCdk13+PE, *p = 0.0151 for siCdk13 (Ser7P): scr vs PE, *p = 0.0232 for siCdk13 (Ser7P): PE vs siCdk13 + PE. Experiments were repeated three independent times with similar results. **(H-K)** Quantification of expression levels of indicated genes from real-time PCR (n=6). **p = 0.0021 for Cdk12: scr vs siTKD, **p = 0.0016 for Cdk12: PE vs siTKD + PE. Data are shown as means ± SEM unless otherwise noted. One-way ANOVA with Tukey's multiple comparisons test was used for all statistical analyses. Exact p values are noted when possible. Source data are provided as a Source Data file. *$p < 0.05$, **$p < 0.01$, ***$p < 0.001$, ****$p < 0.0001$, not significant (n.s., $p > 0.05$) for all indicated comparisons.

Supplementary Figure 4

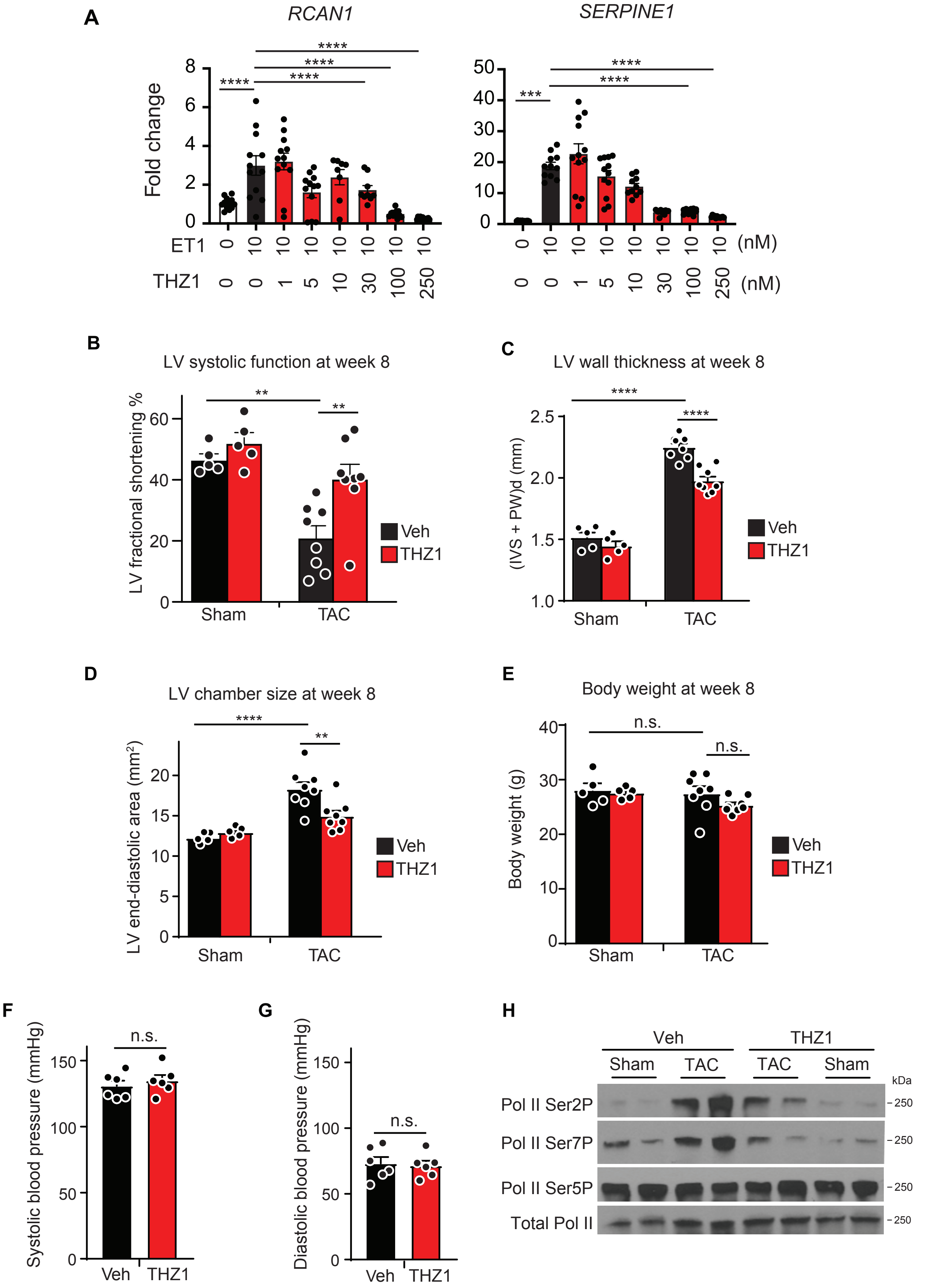

Supplementary Figure 4. Effects of THZ1 on hypertrophic marker gene expression in hiPSC-CM and physiological parameters in mice. (A) Expression levels of representative genes (*SERPINE1* and *RCAN1*) in hiPSC-CM with indicated treatments (n=12 independent samples for RCAN1: baseline, ET1, THZ1-1nM, THZ1-5nM, THZ1-100nM, THZ1-250nM, n=8 independent samples for RCAN1: THZ1-10nM, n=9 independent samples for RCAN1: THZ1-30nM, n=12 independent samples for SERPINE1: baseline, THZ1-1nM, THZ1-5nM, THZ1-100nM, THZ1-250nM, n=11 independent samples for SERPINE1: ET1, n=10 independent samples for SERPINE1: THZ1-10nM, n=9 independent samples for SERPINE1: THZ1-30nM). ***p = 0.0002. **(B)** Two-dimensional echocardiographic LV area fractional shortening at week 8 post-TAC (n=5 sham; n=8 TAC). **p = 0.0018 for Sham-Veh vs TAC-Veh, **p = 0.0064 for TAC-Veh vs TAC-THZ1. **(C)** Wall thickness as expressed as sum of interventricular septum (IVS) and posterior wall (PW) thickness at week 8 post-TAC (n=5 sham; n=8 TAC). **p = 0.0064 **(D)** Left ventricular (LV) end-diastolic area at mid-papillary muscle level at week 8 post-TAC (n=5 sham; n=8 TAC). **(E)** Body weight at week 8 post TAC (n=5 sham; n=8 TAC). **(F)** Systolic blood pressure in mice treated chronically with THZ1 (n=6). **(G)** Diastolic blood pressure in mice treated chronically with THZ1 (n=6). **(H)** Representative Western blot of heart ventricle tissue with indicated treatment for indicated targets. Samples were harvested 60 days after TAC. Pol II phosphoform and total Pol II were each probed from a separately run gel and membrane, with each lane loaded with an equivalent amount of total protein and lysate volume, derived from a common master stock of protein lysate for each designated condition. Experiment was repeated three independent times with similar results. ***p* < 0.01, ****p < 0.0001, not significant (n.s., *p* > 0.05) for indicated comparisons. Data are shown as means ± SEM unless otherwise noted. One-way ANOVA with Tukey's multiple comparisons test was used for all statistical analyses. Exact p values are noted when possible. Source data are provided as a Source Data file.

Supplementary Figure 5

A

Category	Term	% List	FDR (%)
UP_KEYWORDS	Extracellular matrix	13.70	5.91E-32
KEGG_PATHWAY	mmu04151:PI3K-Akt signaling pathway	4.96	1.54E+00
GOTERM_MF_DIRECT	GO:0005178~integrin binding	3.79	3.08E-03
GOTERM_MF_DIRECT	GO:0008083~growth factor activity	4.66	4.19E-05
INTERPRO	IPR015615:Transforming growth factor-beta-related	1.75	7.69E-02
GOTERM_MF_DIRECT	GO:0005125~cytokine activity	3.21	4.22E-01
KEGG_PATHWAY	mmu04350:TGF-beta signaling pathway	2.33	2.20E+00
GOTERM_BP_DIRECT	GO:0030199~collagen fibril organization	3.21	1.33E-05
GOTERM_BP_DIRECT	GO:0001501~skeletal system development	2.92	2.35E-01
INTERPRO	metalloproteinases-like, OB-fold	1.46	2.10E+00

B

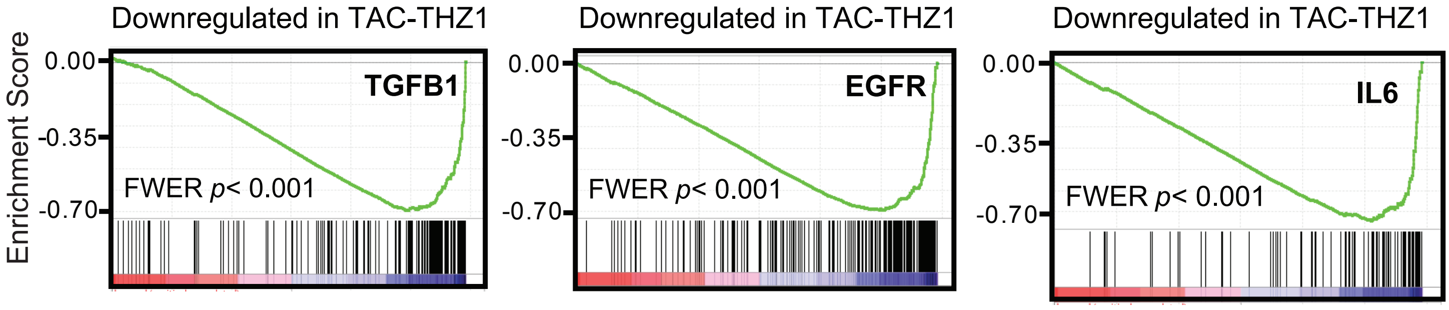

Supplementary Figure 5. THZ1 inhibits specific gene programs in the adult mouse heart. (A) Gene ontology analysis using DAVID for the set of 401 genes that were TAC–inducible and suppressed by THZ1. A false discovery rate (FDR; shown as percent) of < 5% was considered statistically significant. **(B)** Gene set enrichment analysis (GSEA) showing down-regulation of TGFB1, IL6 and EGFR signatures in the set of genes that were upregulated by TAC and suppressed by THZ1.

Supplementary Figure 6

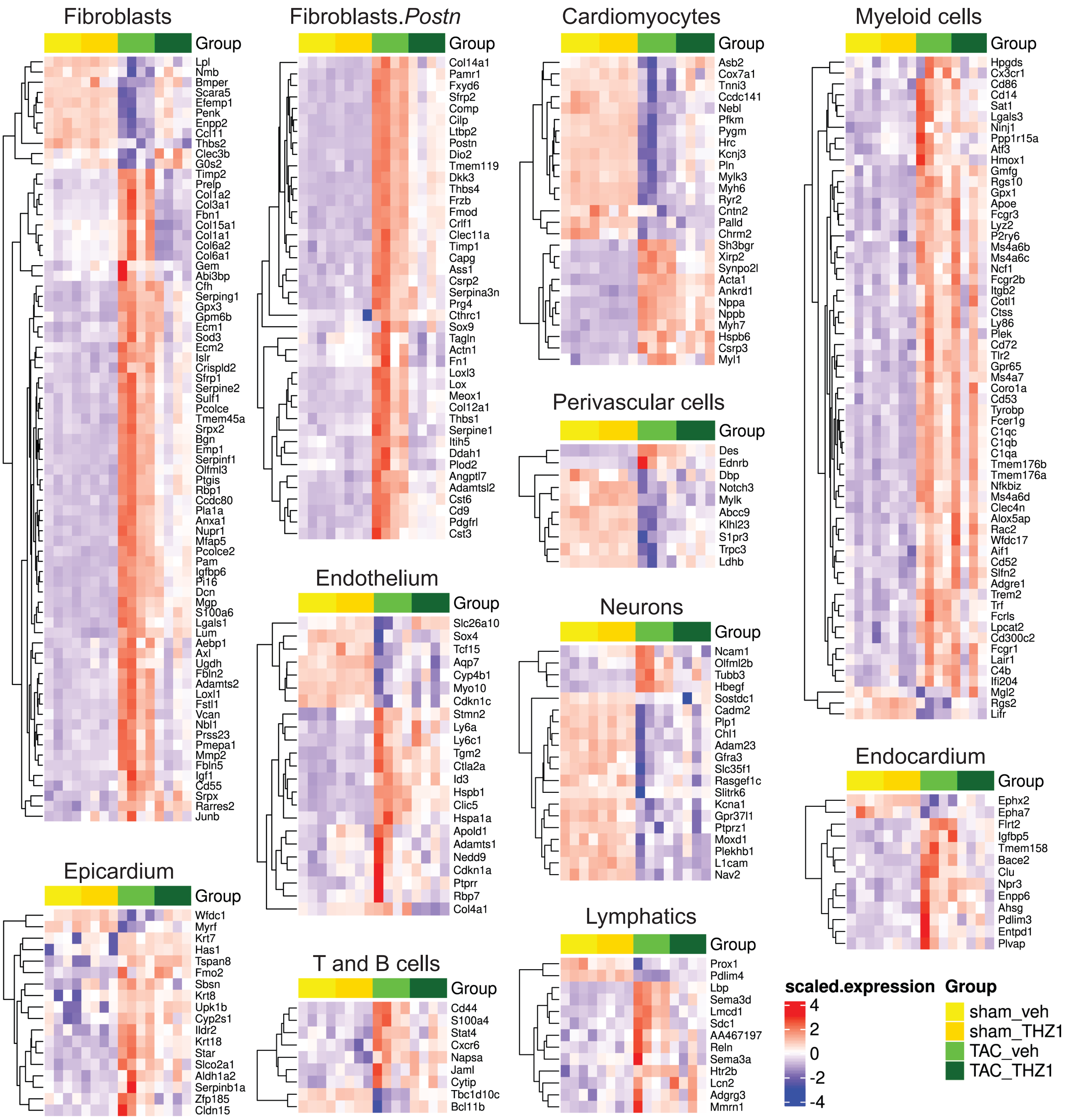

Supplementary Figure 6. Differential gene expression from bulk RNA-seq for TAC vs. sham conditions, binned into indicated cellular compartments (related to Figure 4F-G.). Gene signatures of various cellular compartments determined by curating published single cell RNA-seq datasets from the adult mouse heart. Heatmaps show the expression of genes differentially expressed in TAC-veh vs. sham-veh for all four experimental conditions (sham-veh, sham-THZ1, TAC-veh, TAC-THZ1). Heatmaps are generated for each of the following cellular compartments: fibroblasts, Periostin-positive fibroblasts (Fibroblasts.*Postn*), cardiomyocytes, endothelium, myeloid cells, endocardium, perivascular cells, epicardium, T cells, B cells, lymphatics, and cardiac neurons (fold change less than 0.67-fold or more than 1.5-fold, Benjamini-Hochberg adjusted $p < 0.05$).

Uncropped Blots – Supplementary Figure 1E

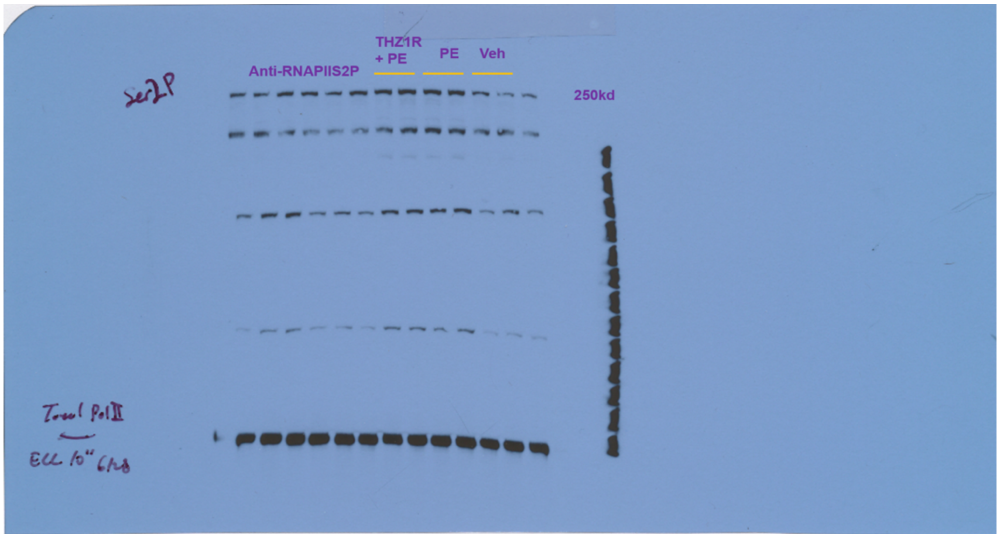

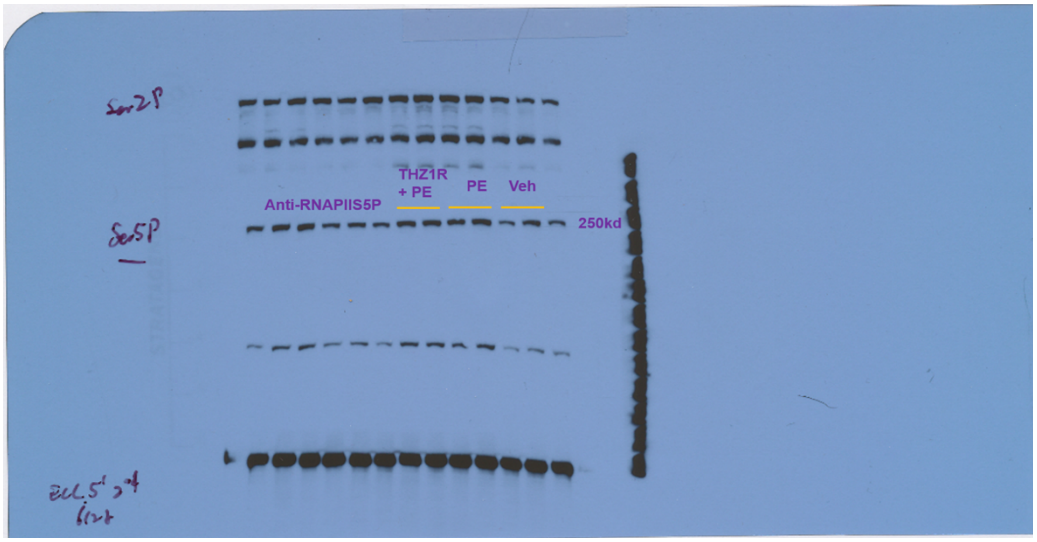

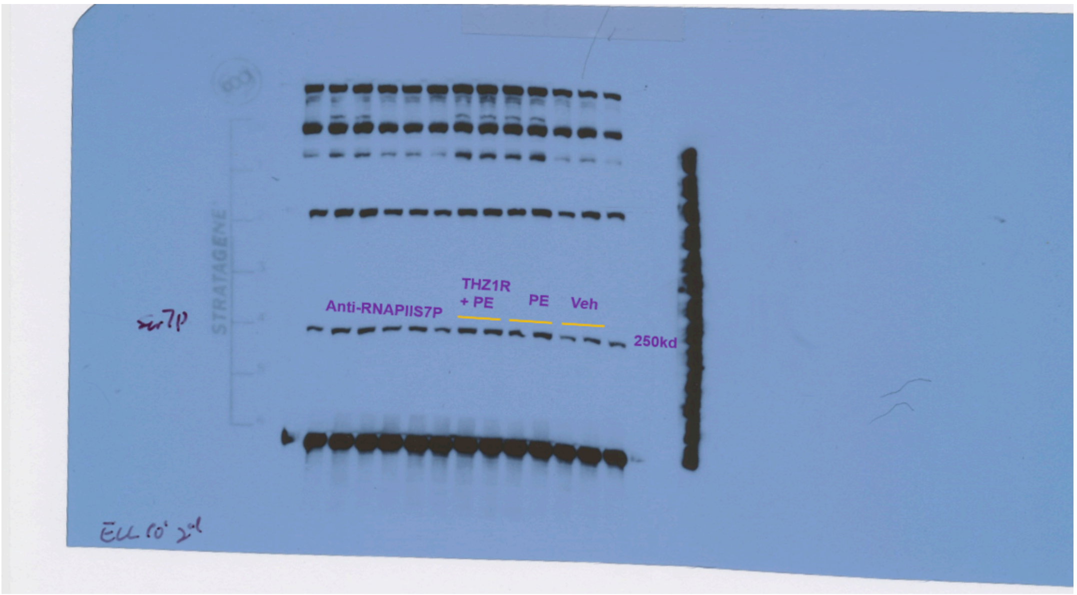

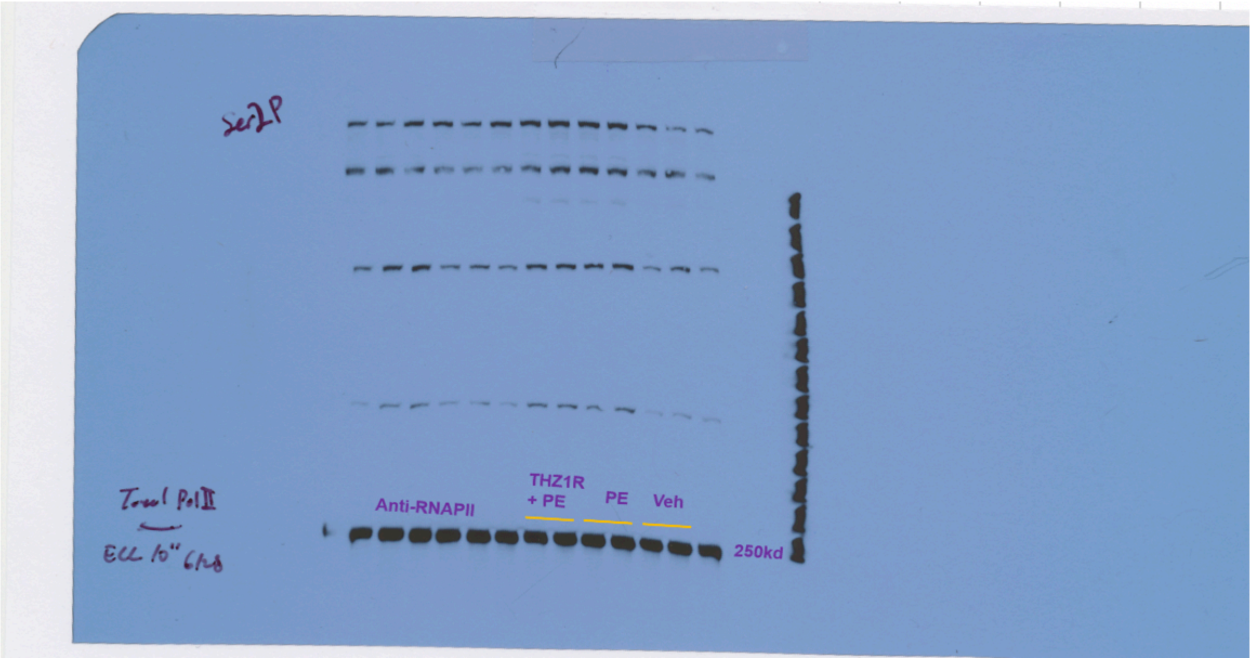
Ser2P
Anti-RNAPII
THZ1R
+ PE
PE
Veh
250kd
Total Pol II
ECL 10" 6/28

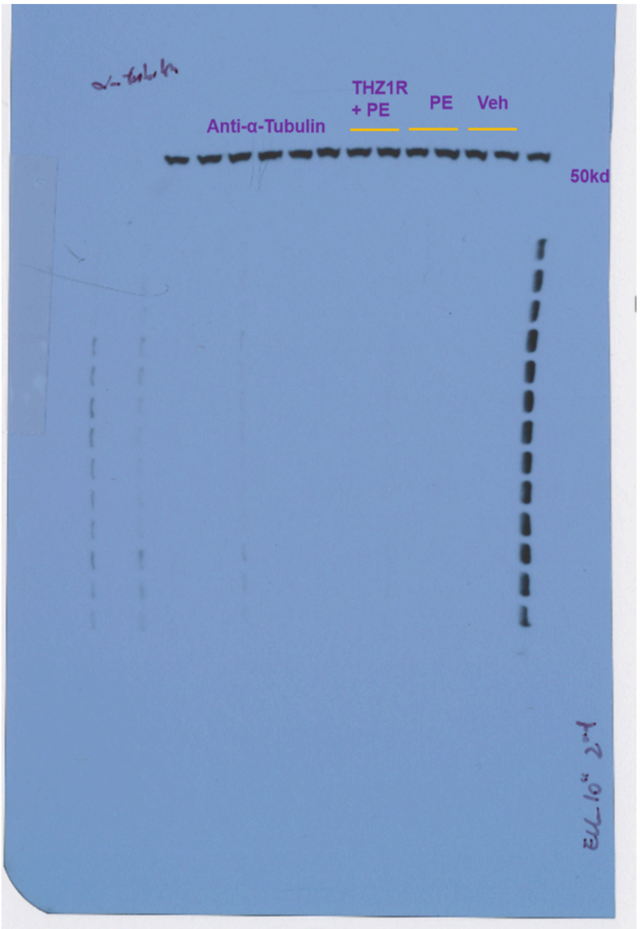
α-tubulin
Anti-α-Tubulin
THZ1R
+ PE
PE
Veh
50kd

Uncropped blots – Supplementary Figure 3C

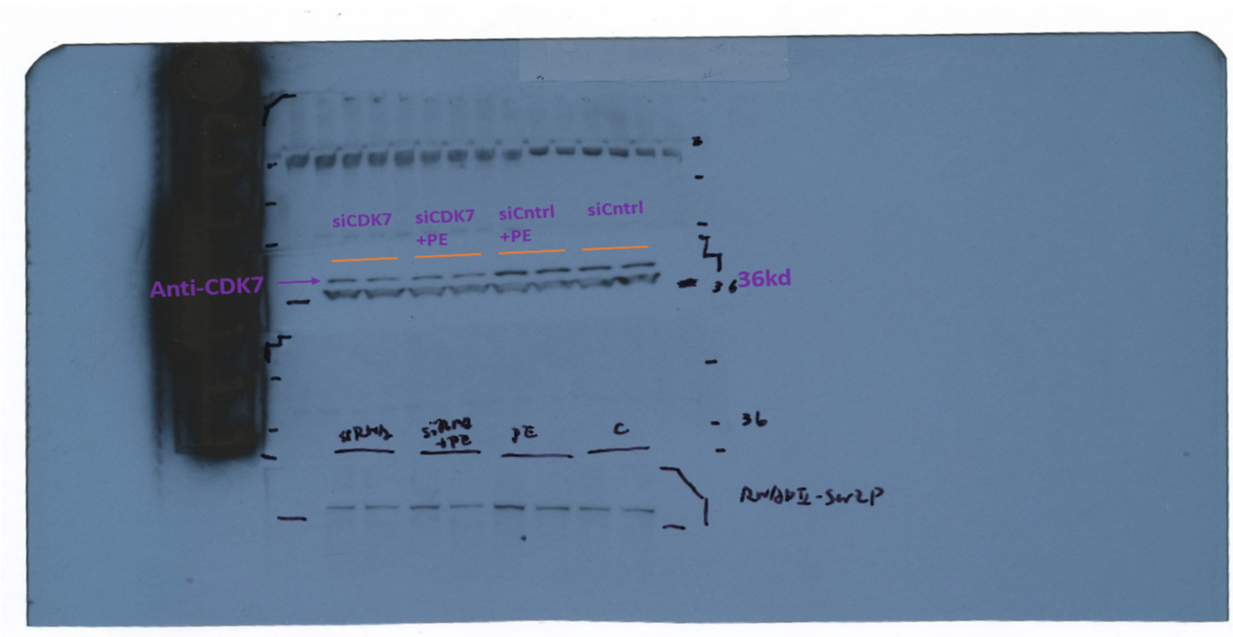

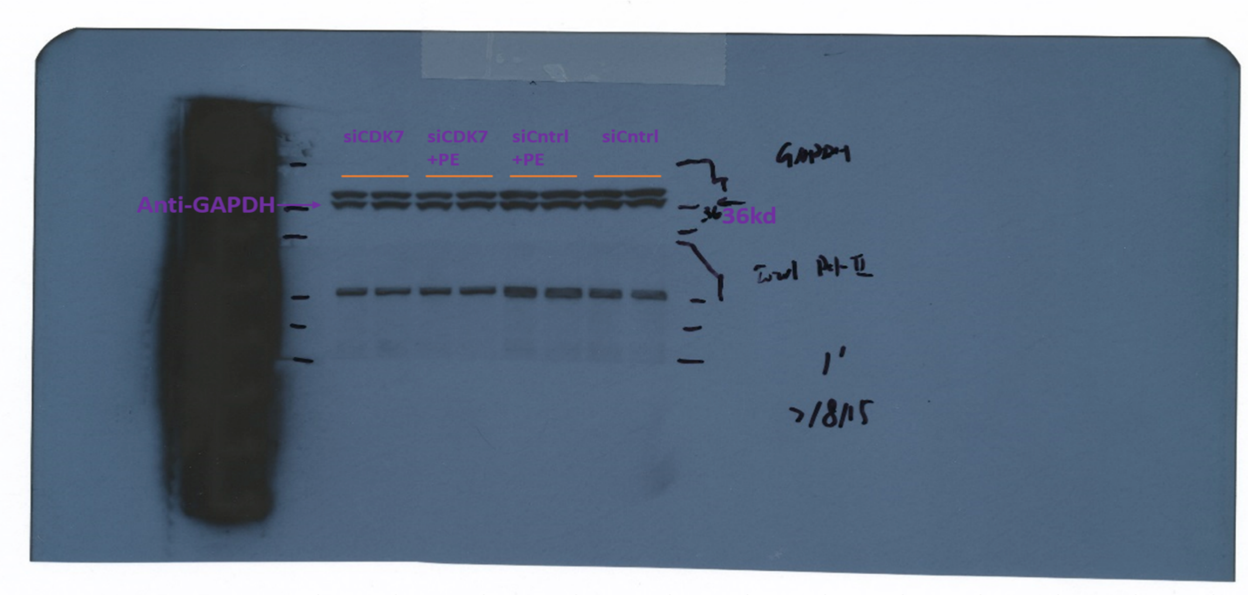

Uncropped blots – Supplementary Figure 3E

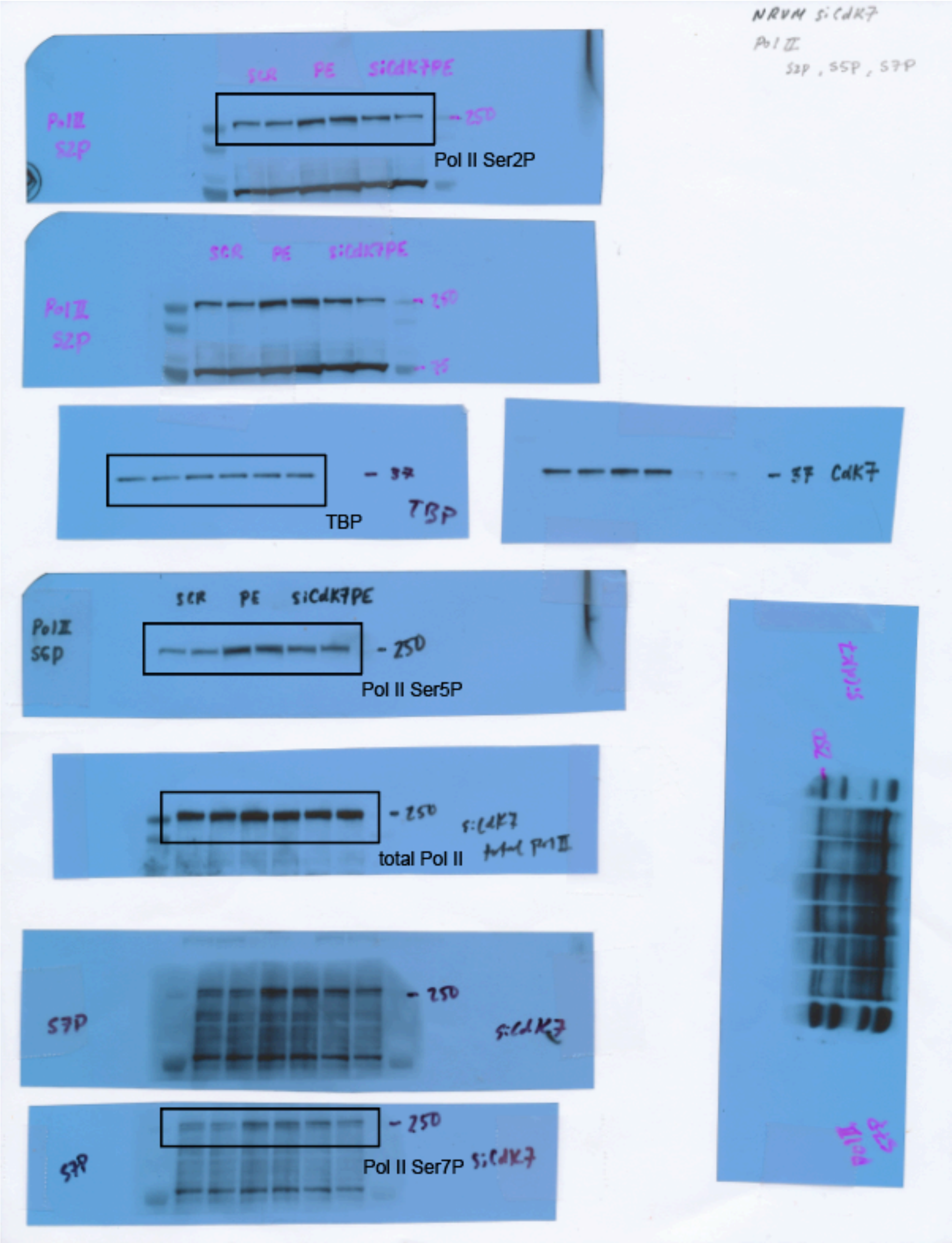

Uncropped blots – Supplementary Figure 3F

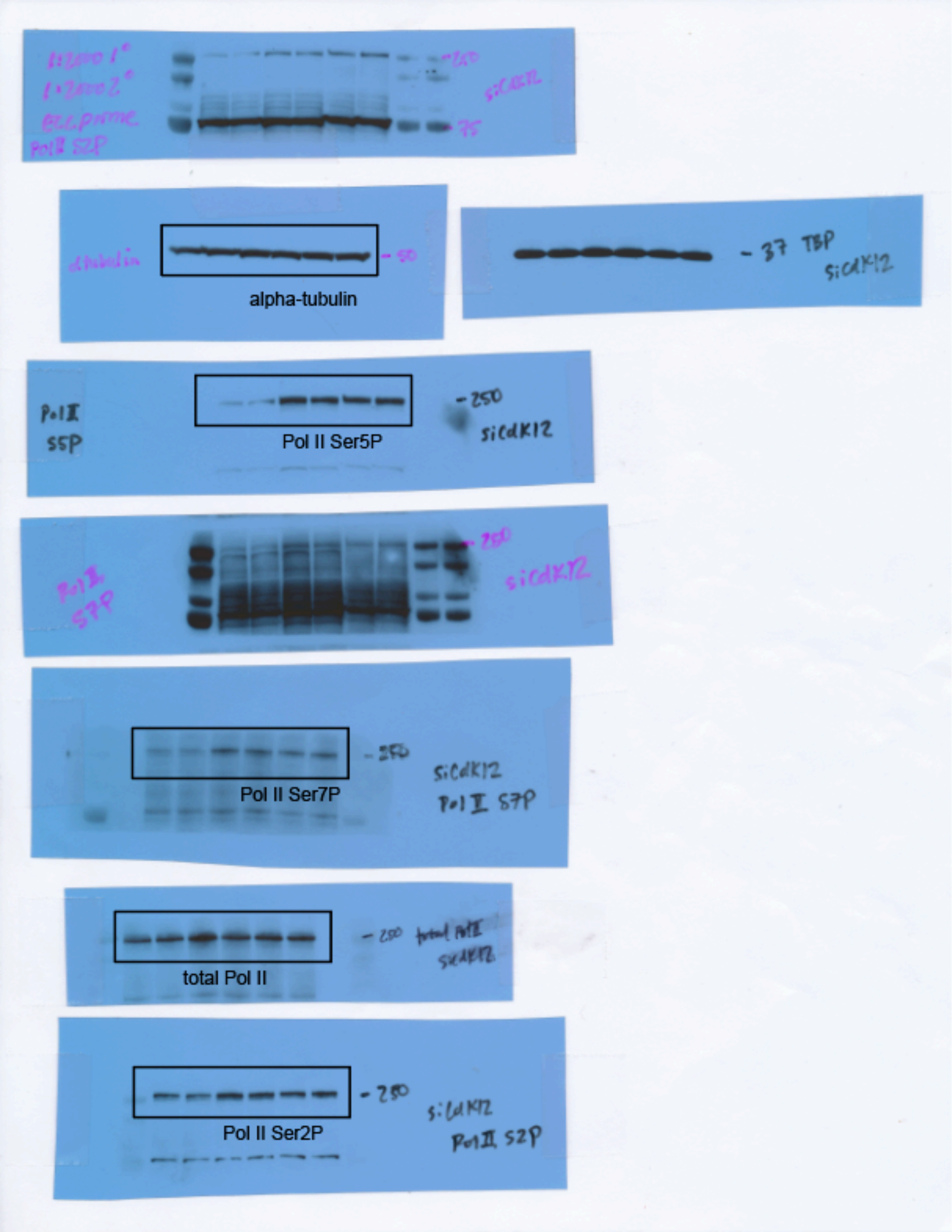

Uncropped blots – Supplementary Figure 3G

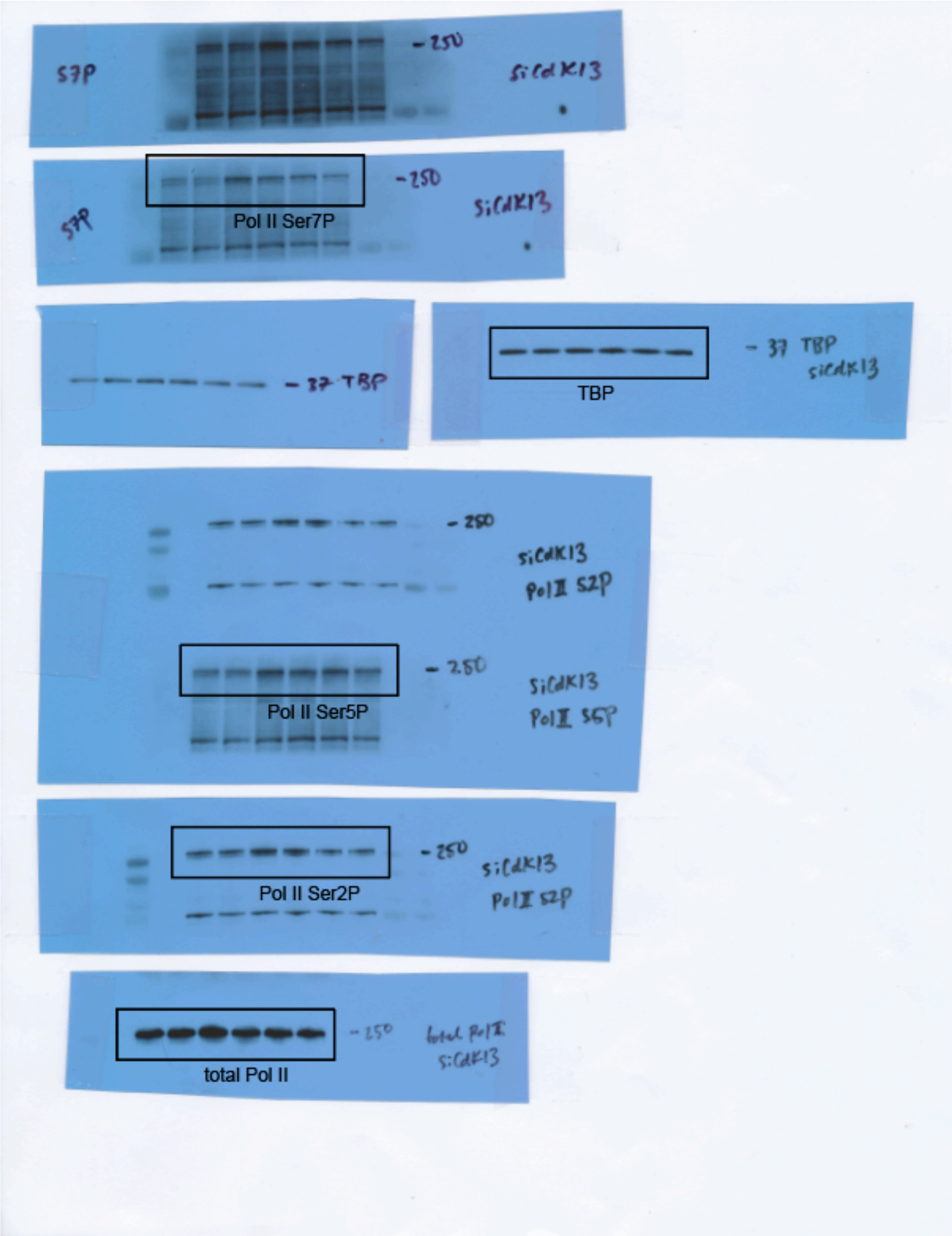

Uncropped blots – Supplementary Figure 4H

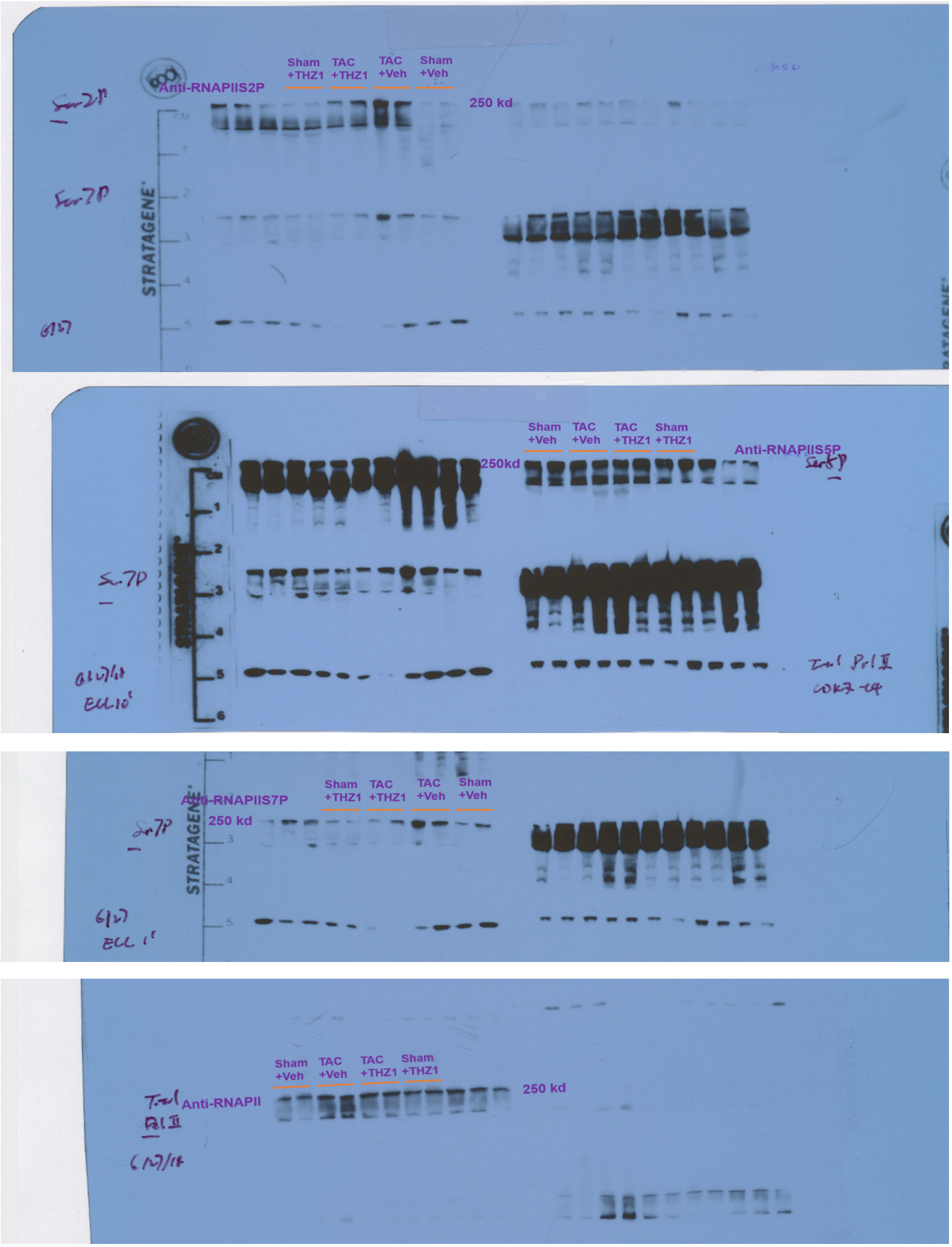

Supplement: Supplementary file 1 — Supplementary information [file 41467_2022_31541_MOESM1_ESM.pdf]
