# Supplementary material for: Targeting transcription in heart failure via CDK7/12/13 inhibition
Source: Nat Commun. 2022 Jul 27;13:4345. doi: 10.1038/s41467-022-31541-8 (PMC9329381; doi:10.1038/s41467-022-31541-8)
Supplement: Supplementary file 3 — Description of Additional Supplementary Files [file 41467_2022_31541_MOESM3_ESM.docx]

**Description of Additional Supplementary Files**

**Supplementary Data 1: Bulk RNA-seq data from NRVM.** Excel sheet of differential gene expression analysis in NRVM (output of DESeq2). Groups compared are untreated (baseline; group 1A), PE stimulated (group 1B), and PE + THZ1 (group 1C). Related to Figure 2.

**Supplementary Data 2:** **Gene ontology analysis of differentially expressed genes in NRVM RNA-seq.** Output from DAVID of gene ontology from each of the differentially expressed gene clusters presented in Figure 2B. Related to Figure 2.

**Supplementary Data 3: Bulk RNA-seq data from mouse LV tissue.** Excel sheet of differential gene expression analysis in mouse LV tissue. Groups shown are Sham-Veh (group 3A), sham THZ1 (group 3B), TAC-Veh (group 3C), and TAC-THZ1 (group 3D). Related to Figure 4.

**Supplementary Data 4: List of qRT-PCR primers and TaqMan mastermixes.** qRT-PCR primers used in mouse and rat experiments are shown. For qRT-PCR in human iPSC-derived cardiomyocytes, the Thermo Fisher catalog number for mastermixes containing validated TaqMan primer and probe combinations are shown.
